# Challenges and translational considerations of mesenchymal stem/stromal cell therapy for Parkinson’s disease

**DOI:** 10.1038/s41536-020-00106-y

**Published:** 2020-11-03

**Authors:** Dominika Fričová, Jennifer A. Korchak, Abba C. Zubair

**Affiliations:** 1 Department of Laboratory Medicine and Pathology and Center for Regenerative Medicine, Mayo Clinic, Jacksonville, FL USA; 2 Institute of Neuroimmunology, Slovak Academy of Sciences, Bratislava, Slovak Republic

**Keywords:** Stem-cell research, Parkinson's disease, Translational research, Mesenchymal stem cells, Parkinson's disease

## Abstract

Parkinson’s disease (PD) is the second most common neurodegenerative disease characterized by the progressive loss of dopaminergic neurons in the substantia nigra pars compacta and the presence of Lewy bodies, which gives rise to motor and non-motor symptoms. Unfortunately, current therapeutic strategies for PD merely treat the symptoms of the disease, only temporarily improve the patients’ quality of life, and are not sufficient for completely alleviating the symptoms. Therefore, cell-based therapies have emerged as a novel promising therapeutic approach in PD treatment. Mesenchymal stem/stromal cells (MSCs) have arisen as a leading contender for cell sources due to their regenerative and immunomodulatory capabilities, limited ethical concerns, and low risk of tumor formation. Although several studies have shown that MSCs have the potential to mitigate the neurodegenerative pathology of PD, variabilities in preclinical and clinical trials have resulted in inconsistent therapeutic outcomes. In this review, we strive to highlight the sources of variability in studies using MSCs in PD therapy, including MSC sources, the use of autologous or allogenic MSCs, dose, delivery methods, patient factors, and measures of clinical outcome. Available evidence indicates that while the use of MSCs in PD has largely been promising, conditions need to be standardized so that studies can be effectively compared with one another and experimental designs can be improved upon, such that this body of science can continue to move forward.

## Introduction

Parkinson’s disease (PD) is the second most common neurodegenerative disease with a prevalence of 0.5–1% among people 65–69 years of age, and rising to 1–3% among people of 80 years of age and older^1^. The pathological hallmarks of PD are a progressive and gradual loss of dopaminergic (DA) neurons of the nigrostriatal pathway and in the substantia nigra pars compacta^2^, the presence of neuronal α-synuclein inclusions called Lewy bodies, and neuro-inflammation in various brain regions^3,4^. The loss of DA neurons in the substantia nigra pars compacta results in typical clinical manifestation and the classification of PD as a movement disorder characterized by resting tremor, postural instability, rigidity, and bradykinesia^5,6^. Symptoms of PD usually appear around 55 years of age when roughly 80% of the nigrostriatal DA system is degenerated^7,8^. However, PD pathology extends beyond the nigrostriatal DA pathway. Lewy pathology is also found in the vagus nerve, spinal cord, sympathetic ganglia, and cardiac and myenteric plexuses^9,10^. This leads to a number of secondary motor and non-motor symptoms^9,11^ such as neuropsychiatric disorders (anxiety and depression), autonomic dysfunction (constipation), sleep abnormalities (insomnia), olfaction and visual disorders, and cognitive decline including dementia^12,13^.

Although the precise mechanism leading to neuronal loss in PD is still unknown, it appears to be multifactorial. The pathogenic mechanisms proposed to play a role in PD include genetic factors, excessive release of oxygen free radicals and oxidative stress, dysfunctional protein degradation, glial dysfunction, lack of trophic factors, inflammation, mitochondrial dysfunction, and accumulation of damaged mitochondria in DA neurons^14,15^.

The main treatments for PD patients include the administration of dopamine precursor l-3,4-dihydroxyphenylalanine (levodopa; l-dopa), dopamine receptor agonists, and inhibitors of endogenous dopamine degradation enzymes (catechol-*O*-methyl transferase and monoamine oxidase B inhibitors), as well as surgical procedures such as deep brain stimulation (DBS)^16^. Pharmacological strategies can restore DA activity and improve the motor symptoms of PD, especially in the early stages of disease. However, their administration does not improve and may even exacerbate non-motor manifestations of PD, such as postural hypotension and neuropsychiatric problems^17,18^. In regard to the most commonly used pharmaceutical therapy, levodopa, prolonged administration results in undesirable side effects such as dyskinesia and neuropsychiatric pathologies including hallucinations and impulsive-compulsive behaviors^19,20^. Similar to pharmaceutical treatment, DBS is effective in controlling the motor symptoms of PD, but does not allay most of the non-motor pathological manifestations^21^. In order to find a cure for PD, various approaches using anti-inflammatory drugs^22^ and neurotrophic factors^23^ are being tested in preclinical models. In line with these increasing efforts to improve the efficacy of PD treatment, cell-based therapy has been raised as a promising alternative approach^24^.

In this review, we describe the evolution of cell therapy in PD, highlight why mesenchymal stem/stromal cells (MSCs) are a promising option in the treatment of this disease, and stress the multitude of potential variabilities that can arise from MSC-based clinical trials in PD.

## Evolution of cell therapy for treatment of PD

Due to the selective degeneration of neurons in PD, cell-based therapies including neuronal transplantation were viewed as compelling therapeutic approaches. The proposed mechanisms of cell transplantation that allow for the mitigation of neurodegeneration and symptoms include neurite outgrowth of grafts, graft innervation, and formation of synaptic connections with the host tissue^25^, the expression of tyrosine hydroxylase, the release of dopamine, and the release of neurotrophic and neuroprotective bioactive molecules from the transplanted cells^26,27^.

Over 50 years ago, the first preclinical studies involving the transplantation of rat and human-derived fetal ventral mesencephalon (hfVM) neuroblasts into rodent brains were conducted^28^. Subsequent studies in animal models of PD demonstrated that transplanted DA neurons obtained from the fetal midbrain were able to integrate into host tissue, release dopamine, and improve motor function. This discovery revolutionized cell-based replacement therapy as a treatment for PD^29^. Based on these encouraging results, several scientific groups conducted open-label clinical trials in PD patients. However, the results were variable, with only moderate to no significant improvement^30,31^. These inconsistencies were largely due to highly variable trial design in addition to technical difficulties including cell type variability in tissue grafts, phenotypic instability after passaging, and poor proliferation and survival in the brain after grafting^32,33^. Further challenges in the use of hfVM neuroblasts in PD treatment included the host immune response to an allogenic graft, ethical concerns regarding the use of fetal tissue, and the potential for malignant transformation^34,35^. Due to these limitations of hfVM neuroblast-based treatment, the focus has shifted to an alternative source of cells for regenerative therapies and transplantation in PD. Recently, enormous progress has been made in the field, and several different strategies and cell sources have been tested for their potential in PD treatment including human embryonic stem cells, human induced pluripotent stem cells (hiPSCs), human neural stem cells, hiPSC-derived neural stem cells, and human MSCs. The concerns and the advantages of different stem cells sources for PD treatment are included in Table 1.

**Table 1:** Comparison of different sources of cells for cell therapy.

	hESCs	iPSCs	hNSCs	iPSC-derived hNSCs	MSCs	iPSC-derived MSCs
Ethical concerns	!	✓	!	✓	✓	✓
Genomic stability	!	!	✓	!	✓	!
Risk of tumor formation	!	!	✓	!	✓	!
Allogenic and autologous source available	!	✓	!	✓	✓	✓
Risk of immunological rejection	!	✓	!	✓	✓	✓
Donor-age-related issues	✓	!	✓	!	!	✓

✓ safe/advantage, ! concerns/disadvantage.

## The potential of hMSC-based therapy for PD treatment

Researchers with interest in PD have focused their attention on cell-based therapies using MSCs due to their widespread availability in the body^36^, proliferative abilities, and immunomodulatory capabilities^37^. The therapeutic potential of MSCs involves several mechanisms. Human MSCs are able to secrete protective neurotrophic factors, anti-apoptotic factors, growth factors, and cytokines (vascular endothelial growth factor (VEGF), hepatocyte growth factor (HGF), insulin-like growth factor 1 (IGF-1), brain-derived neurotrophic factor (BDNF), beta-nerve growth factor (β-NGF), transforming growth factor-β (TGF-β), fibroblast growth factor 2 (FGF2), and glial cell-derived neurotrophic factor (GDNF)) into a damaged, inflamed area^38^. In this way, MSCs could promote repair through the production of anti-inflammatory cytokines such as interleukin (IL)-10 and TGF-β^39^ in addition to the inhibition of pro-inflammatory cytokines (tumor necrosis factor-α, interferon gamma (IFN-γ), and IL-1 β), which have been identified to be released in the brains of PD patients^40,41^. MSCs also mediate hematopoiesis regulation and tissue regeneration through their paracrine signaling and multipotency, respectively^42^. Moreover, MSCs have the potential to be differentiated into a neural lineage, including DA neuron precursors^43,44^. However, it remains unclear whether undifferentiated MSCs or MSCs that have undergone neuronal differentiation are able to integrate into host neural circuits and create new synaptic connections with host neurons^45,46^. Recent findings indicating that MSCs can transfer mitochondria to damaged tissue represent another intriguing mechanism that could be beneficial in PD therapy due to the proposed central role of damaged mitochondria in neurodegeneration of DA neurons^47^. MSC therapy has been shown to be safe with no increased risk of neoplastic transformation (Table 1)^48^.

## The challenges of hMSC cell-based therapies for PD treatment

Previous clinical studies using MSCs in the treatment of PD in humans have provided promising preliminary data. In an open-label study in 2010, autologous bone marrow (BM)-derived MSCs with a dose of 10^6^ cells per kilogram body weight were stereotactically administered unilaterally into the sublateral ventricular zone in seven patients with PD^49^. Three patients were reported to have improved PD symptoms. In 2012, the same research group started another open-label study using allogeneic BM-derived MSCs with a dose of 2 × 10^6^ cells per kilogram of body weight and stereotactic administration bilaterally into the sublateral ventricular zone into eight patients with PD and eight with advanced symptoms of PD recognized as “PD plus”^50^. The group reported persistent improvement of symptoms in PD patients and transient improvement of symptoms in “PD plus” patients.

Currently, seven clinical trials using MSCs for PD treatment are in progress with highly variable set up (Table 2). There is a distinct lack of consistency between clinical trials, such that these studies are difficult to compare with one another in order to pinpoint what needs to be improved upon in future studies. In the following sections, we highlight the sources of variability in MSC-based PD therapy in an effort to draw attention to the need for increased standardization in this field.

**Table 2:** Clinical studies involving MSC therapy in Clinicaltrials.gov website.

Study Title	Source of MSCs	Autologous or Allogenic Transplant	Dose Delivery	PD Pathology	Age	Outcome Measures	Identifier and Phase	Status as of January 2020
Study to assess the safety and effects of autologous adipose-derived SVF cells in patients with Parkinson’s disease	Adipose tissue-derived MSCs	Autologous	Delivered to vertebral artery and intravenous administration	Current diagnosis of PD with motor complications	18–80 years	Change in motor function Changes in mental state	NCT01453803 Phase 1/2	Withdrawn
Outcomes data of adipose stem cells to treat Parkinson’s disease	Adipose tissue-derived MSCs	Autologous	Not provided	Idiopathic PD	18 years and older	Change from baseline in overall quality of life	NCT02184546	Active, not recruiting
Use of mesenchymal stem cells (MSCs) differentiated into neural stem cells (NSCs) in people with Parkinson’s (PD).	Umbilical cord-derived MSCs	Allogenic	Intrathecally and intravenously	Diagnosis of PD for between 1 to 7 years	20–75 years	Tractography Blood-based biomarkers Cerebrospinal fluid-based biomarkers	NCT03684122 Phase 1/2	Recruiting
Allogeneic bone marrow-derived mesenchymal stem cell therapy for idiopathic Parkinson’s disease	Bone marrow-derived MSCs	Allogenic	Intravenously at one of four doses: 1 × 10^6^ MSC/kg, 3 × 10^6^ MSC/kg, 6 × 10^6^ MSC/kg, or 10 × 10^6^ MSC/kg	Idiopathic PD	45–70 years	Change in motor function Change in disability. Functional magnetic resonance imaging or diffusion tensor imaging Change in quality of life Change in cognitive function Change in immunologic response Change in suicidal ideation	NCT02611167 Phase 1/2	Completed
Mesenchymal stem cells transplantation to patients with Parkinson’s disease	Bone marrow-derived MSCs	Autologous	Intravenous administration of up to 6 × 10^5^ MSCs/kg, once a week for 4 weeks	Diagnosis of PD for more than 2 years Motor complications despite optimized levodopa treatment PD of Stage 2–2.5 3 or 4 of Hoehn-Yahr staging	30–65 years	Number of participants with adverse events Effect assessment	NCT01446614 Phase 1/2	Unknown
Umbilical cord-derived mesenchymal stem cells therapy in Parkinson’s disease	Umbilical cord-derived MSCs	Allogenic	Intravenous administration of 10–20 × 10^6^ MSCs once a week for 3 weeks	PD	40–60 years	Change in motor function Changes in mental state	NCT03550183 Phase 1	Recruiting
Individual patient expanded access IND of hope biosciences autologous adipose-derived mesenchymal stem cells for Parkinson’s disease	Adipose tissue-derived MSCs	Autologous	Intravenous administration of 2 × 10^8^ MSCs 2 weeks apart for two infusions and then monthly for 6 additional infusions, 8 infusions total	PD	18 years and older	Change in motor function Change from baseline in overall quality of life Change in immunologic response	NCT04064983	Expanded access no longer available

*PD* Parkinson’s disease, *MSCs* mesenchymal stromal/stem cells, *IND* Investigational New Drug, *NCSs* neural stem cells, *SVF* stromal vascular fraction.

### PD patient: selection and outcome measures

One source of variability in the studies is the PD classification and selection of patients for the study. To date, tests allowing for the diagnosis of PD in early stages are missing. The more precise diagnosis of PD is based on the presence of substantia nigra pars compacta degeneration and Lewy pathology found during the post-mortem pathological examination. Therefore, the current diagnosis of PD is based on the symptoms that arise in the later stages of the disease, when approximately 80% of DA neurons have already been damaged.

Classification and staging of PD vary and can lead to a high degree of heterogeneity in defined PD groups. Nevertheless, classification and prediction of disease progression has significant consequences for the selection of therapeutic strategies and the potential success of treatments. Currently, clinical studies focused on MSC-mediated PD treatment have mainly used age and time from diagnosis as inclusion or exclusion criteria. These studies also vary in the outcome measures for the evaluation of MSC treatment success (Table 2), although strides have recently been made to propose a global consensus of outcome measures for PD^51^. Moreover, these scales, which include the Hoehn and Yahr scale^52^, the Unified Parkinson’s Disease Rating Scale (UPDRS)^53^, the Movement Disorder Society-sponsored revision of the UPDRS^54^, NMS-Quest^55^, and the physician-assisted non-motor symptoms scale^56^, are not only used for selection of the patient but also for measuring the clinical improvements. More objective selection criteria and improvement measures might include functional magentic resonance imaging, magnetic resonance tractography, and blood and cerebral fluid biomarkers^57,58^. Another important question arising from measuring the outcomes is the timing of follow-up. Thus, the use of more unified clinical groups as well as outcome measures is crucial for the successful assignment of cell therapy treatment strategies.

### Donor: autologous versus allogenic

MSC-mediated treatment offers both options: autologous and allogenic transplantation. Autologous MSCs are mainly isolated from adipose tissue (AD) and BM. Variability in autologous MSC therapy between patients can stem from different aspiration techniques during harvesting, which can influence cell yield, viability, and differentiation potential^59,60^. Autologous MSCs are more likely to garner harvesting variabilities than allogenic MSCs, due to the need to harvest MSCs from each patient rather than having one source of expanded MSCs for experimental use. Some studies in AD-derived MSCs have found a negative correlation between donor age and cell proliferation of the MSCs^61^. Similarly, the yield of MSCs from BM aspirate has been found to have a negative correlation with age^62^. Furthermore, a study using immortalized AD-derived MSCs from PD patients have demonstrated significant mitochondrial dysfunction in the MSCs as compared to the immortalized MSCs of non-PD patients^63^. Additionally, several studies have proposed that PD pathology might limit the regenerative capacity of autologous MSCs due to their autophagy and decreased mitochondrial functions^64^. This raises concerns regarding the potential efficacy of PD patient-derived MSCs. Furthermore, both pathological and clinical studies have shown that PD pathology affects not only the central nervous system (CNS) but spans through several organs, which could impact the effectiveness of autologous MSC treatment. In addition to the systemic nature of PD, the age of the patient and the systemic effect of PD medication both have important implications for autologous MSC treatment and should be taken into consideration^65^.

Allogenic MSCs can be isolated from umbilical cord (UC) in addition to BM and AD. The advantages of allogenic MSC transplants include increased timely availability of cells when needed, reduced overall cost, decreased harvesting variability, and the opportunity to conduct more thorough cell quality assessments^66^. In contrast to autologous MSC treatment, trials involving allogenic MSCs are able to treat patients using MSCs that had undergone extensive quality-assurance measures to mitigate any batch stability or genome issues. Although cryopreservation is applicable to both autologous and allogenic MSCs, variations in freezing technique, composition of freezing media, viability and therapeutic effectiveness of MSCs after thawing represent additional sources of variation among clinical studies^67^. Another factor to consider in allogenic MSC transplantation is the potential for immune rejection^66,68^. This raised the question of whether donor human leukocyte antigen (HLA) should be matched with patients or patients should undergo immunosuppression before and/or after MSC transplantation. However, HLA matching would entail the need to have more donors, which would increase time, cost, and harvesting variables^69^. Additionally, immunosuppression increases the risk of cancer, infection, cardiovascular and metabolic disease, and immune dysregulation, all of which could negatively impact the lives of the patients^70^. Furthermore, multi-dose regimens have the potential to accelerate the clearance of MSCs due to the boosted allogenic memory-response^71^. Recent studies have suggested that the immunogenicity of MSCs have no significant adverse impact on the engraftment of MSCs in wound healing^72^. In general, it is believed that MSCs express a low level of HLA antigen^73^. However, studies have shown that major HLA class II molecules could be increased during in vitro expansion, which highlights the importance of using low-passage MSCs^68^. Therefore, while HLA matching may not be necessary, administering HLA-matched MSCs may prolong the survival of the MSCs in vivo. Even though allogenic MSC therapy has been shown to be well tolerated with minimal side effects, there is still concern for the risk of transmitting blood-borne pathogens, and immune rejection, which can lead to the elimination of MSCs and thus potentially lead to a reduced therapeutic effect^66,74^. Furthermore, an important limitation to the clinical application of both autologous and allogenic MSCs is the potential for their spontaneous differentiation into undesirable cell lineages and tissue types^75^.

### Donor: sources of MSCs

The primary sources of MSCs are BM aspirate, AD, and UC^76,77^. Considerations must be made regarding the selection of a source. One factor to consider for is the ease and efficiency of harvesting. The harvesting method itself can introduce experimental variability, which has the potential to significantly affect the therapeutic effectiveness of MSCs. Variability can stem from different injection sites within the same sources, or aspiration techniques, which can influence cell yield, viability, and differentiation potential^59,60^. Compared with AD and UC, harvesting BM is the most invasive and can cause the most pain and infection risk^78,79^. BM aspirate contains only 0.001–0.01% MSCs in the overall cell population, which translates to roughly 60–600 cells/mL of aspirate^76^. Considering that only a limited volume of aspirate can be withdrawn, this means that there will typically be an intensive culturing process to expand the MSCs, especially if the cells are to be used allogenically^76,79^. AD can be harvested from lipid waste generated from lipectomy, lipoplasty, and liposuction. AD can also be harvested from a small area under local anesthesia, making this procedure much less invasive and hazardous than BM harvesting and therefore arguably a better source for autologous use^79,80^. Furthermore, an AD harvest obtains a 500 times greater yield of MSCs than an equivalent amount of BM aspirate^81,82^. Compared to BM and AD, UC harvesting is the least invasive and poses no ethical challenges due to the UC tissue being derived aseptically from cesarean sections^83,84^. Additionally, due to the nature of UC, there are no age-related problems, which can cause issues in adult MSC isolations. Furthermore, a large amount of MSCs can be derived from one UC, which can minimize the need to extensively expand the cells for allogenic use^84^. UC tissue can also be cryopreserved after harvesting, allowing for the potential to isolate MSCs as needed. However, the isolation of living MSCs from thawed, previously cryopreserved tissue is not always possible due to the differential proximity of the MSCs to the cryoprotectant^85^.

In addition to harvesting considerations, there are also proliferation differences between sources that need to be considered. It has been found that UC-derived MSCs proliferate faster than AD-derived MSCs, and AD-derived MSCs proliferate faster than BM-derived MSCs^83,86^. UC-derived MSCs on average have the shortest doubling time at 24 h^87^, compared to AD-derived MSCs which have a doubling time of 40 h, and BM-derived MSCs which have a doubling time of 60 h^79,88^. UC-derived MSCs may be more highly proliferative due to UC-derived MSCs not being inhibited by cell-to-cell contact, which allows them to continue to proliferate even after reaching confluence^89^. Additionally, differences in proliferative abilities may be impacted by the effects of senescence on MSCs. BM-derived MSCs have been found to have senescence landmarks starting at passage 7 (ref. ^90^), and AD-derived MSCs starting at passage 8 (ref. ^88^), which can influence the therapeutic effectiveness, number, maximum lifespan, and differentiation abilities of the cells. In contrast, UC-derived MSCs can easily be expanded over passage 16 without any landmarks of senescence and no karyotype instability or variations in morphology^91^.

Different sources of MSCs have also been found to have differences in their immunological characteristics, as well as have different paracrine signaling and immune modulation abilities^92^. By definition, MSCs should not have HLA-DR surface markers. However, studies have shown that BM and AD-derived MSCs express HLA-DR surface markers in response to IFN-γ, while UC-derived MSCs do not^93^. Despite these findings, a study in BM-derived MSCs have demonstrated that HLA-DR surface marker expression appears to be random, and their presence had little effect on the immunomodulation, multilineage differentiation, and their allogenic immune response^94^. Another surface marker, CD142, has been linked to thrombosis and is a concern for systemic administration of MSCs. BM-derived MSCs have been shown to have lower levels of CD142 as compared to low-passage AD-derived MSCs^95,96^, which could make BM-derived MSCs more suitable for intravenous MSC delivery and decrease the risk of thrombosis^97^. In addition to differences in surface markers, MSCs from different sources have been found to have varying paracrine functions. BM-derived MSCs have been found to have significant paracrine functions, including the secretion of angiogenic factors, growth factors, and cytokines^98^. It has been found that there is a lower secretion of pro-angiogenic molecules and cytokines in AD-derived MSCs as compared to BM-MSCs, which suggests that AD-derived MSCs might be less suited to reducing inflammation^99^. However, UC-derived MSCs have been found to express more angiogenic, neuroprotective, and neurogenerative factors compared to BM-derived MSCs, which make them an attractive option in PD therapy^100^. Furthermore, UC-derived MSCs have been found to exhibit a 20–800k relative fold change compared to BM-derived MSCs, which exhibit a 50–1000 relative fold change for indoleamine-pyrrole 2,3-dioxygenase (IDO) upregulation following IFN-γ stimulation^69^. IDO suppresses T cell responses, which could help increase immune tolerance to allogenic administration of MSCs^101^.

Another factor that needs to be considered, especially in the treatment of PD, is the impact that the source of the MSCs has on their neuronal differentiation abilities. While BM, AD, and UC-derived MSCs have all been demonstrated to express synaptophysin as evidence of the formation of a synapse^102^, AD-derived MSCs were exhibited to express the highest level of SAP-90 as compared to BM- and UC-derived MSCs, which indicates that AD-derived MSCs may be more likely to form synaptic structures^103^. Likewise, BM-, AD-, and UC-derived MSCs were all capable of expressing NT-3, a neurotrophic factor, but AD-derived MSCs had the highest expression^103^. Furthermore, BM, AD, and UC-derived MSCs were all able to express DA neuron markers in vitro, including nurr1 and tyrosine hydroxylase, which is especially relevant in PD therapy^103,104^.

A potential strategy for a more objective classification of MSCs includes the analysis of their biomarkers. The minimum criteria for defining the phenotype of MSCs includes the expression of CD73, CD90, and CD105, and the lack of expression of CD45, CD34, CD14 or CD11b, CD79a or CD19, and HLA-DR^105^. Besides these markers, MSCs express many other surface markers and secrete various bioactive molecules including proteins, immune-modulating molecules, and microRNAs^106^. Multiple comprehensive transcriptomic and proteomic analyses of human MSCs have revealed different markers that may contribute to the molecular classification of subspecies of MSCs. This could lead to a more targeted approach to MSC therapy, where subspecies of MSCs are applied to specific clinical conditions that their phenotype is most suited to treating^107^. However, accumulating evidence suggests that marker expression of MSCs is not stable in culture conditions, which could make characterizing MSCs based on their markers a challenge^108^. So far, it is not clear if the classification based on novel markers can be applicable to clinical studies, but efforts remain ongoing.

### Route of delivery and other variabilities

There are several routes for MSC administration in PD treatment. One option is a direct stereotactic transplantation into the affected area. In rodent PD models, direct transplantation into the striatum, substantia nigra, or subthalamic nucleus has been shown to be effective when using BM-derived MSCs^109–114^, AD-derived MSCs^115–117^, and UC-derived MSCs^118–121^. In some of the stereotactic transplant studies, undifferentiated MSCs were shown to differentiate into DA neurons in vivo^109,110,118,120^. Other studies used MSCs that were differentiated into neurons^111,113,116,118^, neurotrophic factors-secreting cells^112^, or nestin-positive stem cells^114^. Regardless of the PD rodent model (6-hydroxydopamine, 1-methyl-4-phenyl-1,2,3,6-tetrahydropyridine, or rotenone model), the majority of these experiments were successful, as was measured by behavioral improvement, reduced microglial activation, increased tyrosine hydroxylase immunoreactivity, neuroprotection of DA neurons, and even neurogenesis^117,122^. Interestingly, AD-derived MSCs did not differentiate in vivo to DA neurons in any of the studies that were reviewed^115–117^ (Supplementary Table 1). Although the preclinical data for direct transplantation are promising, this delivery route involves a relatively complex surgical procedure, surgery-related risks, possible post-surgical complications, inconvenient administration of repetitive doses, and high costs^123^.

MSCs have the ability to migrate to injury sites and promote repair, which makes them compelling candidates for systemic administration. Systemic administration of MSCs has been shown to be effective using BM-derived MSCs^124–130^ and AD-derived MSCs^126^. In many of these studies, behavioral improvement, neuroprotective effects, and neurogeneration were observed. However, in one study, the MSCs were not able to transmigrate across the blood–brain barrier without a permeabilizing agent^131^, and in another study, the majority of the MSCs were found to be retained in the lungs rather than in the brain^129^. While some studies have shown that intravenously administered MSCs can enter the brain without the aid of a permeabilizing agent^125,129^, many studies did not include experiments regarding the presence of MSCs in the brain^126,127,130^. The less successful therapeutic outcomes observed via systemic administration of MSCs may be due to the MSCs having difficulty transmigrating across the blood–brain barrier^132,133^.

An alternative route for the treatment of CNS diseases is intranasal administration^132^. Studies in preclinical PD models using intranasal administration have demonstrated the successful delivery of MSCs to the brain with localization of MSCs in the olfactory bulb, cortex, hippocampus, striatum, cerebellum, brain stem, amygdala, hippocampus, and spinal cord even 4.5 months after injection^134^. Furthermore, one study has reported neuroprotective effects, anti-inflammatory effects, and improvements in neurobehavioral tests, indicating that intranasally delivered MSCs are a promising therapeutic option for PD^135^.

Other variabilities include questions regarding the dose, if the injections should be repeated, and, if so, how those injections should be timed. To date, an effective dose of MSCs for PD applications has not been optimized, and likely is different between administration routes. In the reviewed clinical trials for PD, some studies reported doses that ranged from 6 × 10^5^–10 × 10^6^ MSCs per kilogram of the patients’ weight, some reported the total amount of MSCs used per patient and disregarded weight, and some studies did not report dosage at all. A meta-analysis of animal models of PD concluded that a higher dose of MSCs (<1 × 10^6^ versus ≥1 × 10^6^ MSCs) did show a significant difference in effect for limb function but not in rotation behavior^136^, yet it is not known whether there is the same effect in non-human primates or humans. Additionally, the studies that have been conducted in primate models of PD have been largely stereotactic^137^, whereas the majority of clinical trials are reported to use intravenous administration, making it difficult to draw conclusions regarding dosage considerations. Standardization of dosage is necessary to limit variabilities between trials and to gain insight into what aspects needs to be improved.

## Future directions and conclusions

Over the decades, scientists and clinicians have put in a tremendous amount of effort into establishing stem cell therapy as an efficient and feasible treatment for neurological diseases including PD. However, systematic translational use of cell therapy is still somewhat out of reach. In order to improve the efficiency of MSC-mediated therapy, several novel strategies have been tested.

One of these approaches is MSC priming or preconditioning. Cell priming involves the exposure of cells to growth conditions that mimic the in vivo microenvironment of damaged tissue. Studies have shown that MSCs can modulate their cellular signaling in response to primed culture conditions^138^. This pre-activation of intracellular molecular signaling before the transplantation of MSCs may improve their function, survival, and therapeutic efficacy. Several priming approaches have been tested, including priming with inflammatory cytokines or mediators, hypoxia, pharmacological drugs, chemical agents, biomaterials, and different culture conditions^139^. The disadvantage of this approach is the limited consensus in cell manufacturing protocols, which leads to difficulty in providing quality assurance for clinical-grade MSCs^140^.

Recently, cell-free therapy has been investigated as a promising alternative approach to regenerative medicine. Several studies have established that the secretome of MSCs, which includes cytokines, growth factors, and various bioactive molecules, is what mediates their therapeutic properties. MSC-conditioned medium has been shown to have therapeutic potential in cardiovascular disease, osteoarthritis, spinal cord injury, gastric mucosal injury, and colitis^141,142^. One of the components of MSC-conditioned medium is extracellular vesicles (EVs). EVs are nanovesicles that contain numerous types of proteins and RNAs, mediate communication between cells, and regulate various biological processes including immune response, angiogenesis, proliferation, and differentiation^143^. EVs have emerged as a key component in the MSC-mediated therapeutic response in the cardiovascular, neurological, musculoskeletal, and immune systems^144^. The advantage of using EVs isolated from MSC-conditioned media stems from the therapy being a cell-free approach, which requires easier administration and avoids the potentially adverse effects of cell therapy. MSC-derived EVs have been shown to provide therapeutic benefits in the treatment of various CNS disorders including stroke^145^, traumatic brain injury^146^, Alzheimer’s disease, amyotrophic lateral sclerosis, and Huntington’s disease^147^. Moreover, it has been shown that MSC-derived EVs mediate the rescue of DA neurons in rodent PD models^148^. Additionally, the ability of EVs to cross the blood–brain barrier presents an attractive biological vehicle for the delivery of bioactive molecules into the brain^149^. However, EVs may be a part of heterogeneous populations, and their metabolomic and lipidomic profiles have not yet been well characterized. Other limitations of EV isolation and purification involve the procedure itself, which includes variability in the quality of EV preparations, the yield of EVs, and the potential for non-EV contaminants in the preparation^150^. Before using EVs in clinical trials, this approach still needs to be extensively evaluated for safety and efficacy.

In order to improve the generation of homogeneous*,* standardized, high-quality MSCs, the production of MSCs from hiPSCs has been proposed as an unlimited source of cells for therapeutic applications in regenerative medicine. Although hiPSC-derived MSCs meet the criteria for MSCs in terms of marker expression, other criteria such as the potential to differentiate to chondrogenic and adipogenic tissue are reduced compared with BM-MSCs^151^. The safety and efficacy of iPSC-derived MSCs are of paramount importance for successful application in the field of translational regenerative medicine. The major concerns regarding iPSC-derived MSCs include determining a suitable starter cell line^152^ and using a reprogramming strategy that is safe for patients. The viral vector-based strategy for reprogramming might present a potential for tumorigenic transformation^153^. However, recent developments in non-viral based technologies including chemicals, plasmids, and recombinant protein-based approaches might present safer strategies for the generation of iPSC-derived MSCs suitable for use in a clinical setting^154^.

Genome-edited MSCs that over-express or inhibit specific genes represent another challenging yet promising approach to improve the therapeutic properties of MSCs. Specifically for PD, several studies using engineered MSCs that expressed tyrosine hydroxylase^155^, vascular endothelial growth factor^156^, or were transduced to produce increased glial cell-derived neurotrophic factor^157^ or cerebral dopamine neurotrophic factor^158^ have demonstrated positive results in preclinical rodent models. However, viral transduction and genetic modification imparts added safety concerns to cell therapy, which creates additional barriers to clinical testing.

**Fig. 1:**
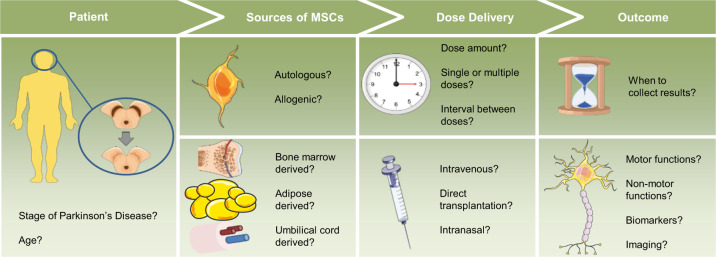
Roadmap of clinical considerations regarding the use of MSCs in PD therapy. Relevant sources of variability in clinical trials for PD include patient factors, MSC sources, dose delivery, and clinical outcomes of therapy. Created using elements of Servier Medical Art by Servier, licensed under CC BY 3.0 (https://smart.servier.com/).

The field of cell-based therapies for PD treatment has faced several challenges. The missing or modest clinical improvement in PD patients treated with MSCs seems to have been the consequence of high variability in clinical trials. In this review, we wanted to stress that allogenic versus autologous transplantation, donor tissue source, culture conditions, PD stage, route of administration, dose, clinical evaluation criteria, and timing of evaluation are sources of variability that can lead to inconsistent results (Fig. 1). Nevertheless, MSCs harbor significant therapeutic potential for the treatment of PD. The advantages of using MSCs for PD therapy include their widespread availability and accessibility, potential for transdifferentiation into neural lineages including DA neurons, immunosuppression in the brain and inhibition of pro-inflammatory cytokines, migratory capacity towards damaged areas, and limited histocompatibility and ethical concerns. The experience gained in previous clinical trials should guide the future directions and emphasize the crucial need for a systematic approach to searching for optimal combinations of conditions in order to achieve reliable and effective treatment designs for PD.

## Supplementary information

Supplementary Information
