# Supplementary material for: Challenges and translational considerations of mesenchymal stem/stromal cell therapy for Parkinson’s disease
Source: NPJ Regen Med. 2020 Nov 3;5:20. doi: 10.1038/s41536-020-00106-y (PMC7641157; doi:10.1038/s41536-020-00106-y)
Supplement: Supplementary file 1 — Supplementary Information [file 41536_2020_106_MOESM1_ESM.pdf]

Supplementary Table 1 Preclinical trials using naïve MSCs in PD rodent models

Delivery route	PD model	MSC type	Auto or Allo	Immunosupression?	Brief Outcome	Ref
Stereotactic Transplant	6-OHDA rat model	BM MSCs	Allo	No	Behavioral improvement<br>Differentiated into dopaminergic neurons *in vivo*	109
	6-OHDA rat model	BM MSCs	Allo	No	Behavioral improvement<br>MSCs *in vivo* differentiation status not reported	111
	6-OHDA rat model	BM MSCs	Allo	Yes	No behavioral improvement<br>MSCs *in vivo* differentiation status not reported	112
	6-OHDA rat model	BM MSCs	Allo	Yes	Behavioral improvement<br>MSCs *in vivo* differentiation status not reported	113
	6-OHDA rat model	BM MSCs	Allo	No	Behavioral improvement<br>MSCs *in vivo* differentiation status not reported	114
	6-OHDA rat model	AD MSCs	Allo	No	No behavioral improvement<br>Working memory test improvements<br>No dopaminergic neuron differentiation detected *in vivo*	115
	6-OHDA rat model	AD MSCs	Allo	No	No behavioral improvement<br>Enhanced endogenous neurogenesis in subventricular zone after 3 days<br>MSCs *in vivo* differentiation status not reported	117
	6-OHDA rat model	AD MSCs	Auto	No	Behavioral improvement<br>No dopaminergic neuron differentiation detected *in vivo*	116
	6-OHDA rat model	AD MSCs	Allo	No	Behavioral improvement<br>MSCs *in vivo* differentiation status not reported	118
	6-OHDA rat model	UC MSCs	Allo	No	Behavioral improvement<br>MSCs *in vivo* differentiation status not reported	119
	6-OHDA rat model	UC MSCs	Allo	No	Behavioral improvement<br>Significantly increased number of dopaminergic neurons in the substantia nigra pars compacta<br>MSCs *in vivo* differentiation status not reported	121
	MPTP rat model	BM MSCs	Allo	No	Behavioral improvement<br>Differentiated into dopaminergic neurons *in vivo*	110
	Rotenone model	UC MSCs	Allo	No	Behavioral improvement<br>Differentiation of MSCs to dopaminergic neurons *in vivo* after 12 months	120

Supplementary Table 1 continued

Delivery route	PD model	MSC type	Auto or Allo	Immunosupression?	Brief Outcome	Ref
Systemic Injection	6-OHDA rat model	BM MSCs	Allo	No	Behavioral improvement<br>MSCs *in vivo* differentiation status not reported	130
	6-OHDA rat model	BM MSCs	Allo	No	Behavioral improvement<br>MSCs *in vivo* differentiation status not reported	129
	6-OHDA rat model	BM MSCs	Allo	No	Behavioral improvement<br>No reduction in neurodegeneration was observed<br>MSCs *in vivo* differentiation status not reported	131
	6-OHDA rat model	AD MSCs	Allo	No	Behavioral improvement<br>Observed rescue of endogenous neurons from cell death<br>MSCs *in vivo* differentiation status not reported	126
	MPTP rat model	BM MSCs	Allo	No	Behavioral effects were not investigated<br>Significant increase in neurogenesis in the substantia nigra<br>No MSC differentiation to dopaminergic neurons *in vivo*, but the MSCs induced differentiation of neural precursor cells into dopaminergic phenotype in the substantia nigra	128
	MPTP rat model	BM MSCs	Allo	No	MSCs reduced the loss of dopaminergic neurons<br>MSCs *in vivo* differentiation status not reported	127
	MPTP rat model	BM MSCs	Allo	No	No behavioral improvement<br>MSCs were found to have reached the brain, but did not observe differentiation to dopaminergic neurons *in vivo*	125
Intranasal Injection	Rotenone model	BM MSCs	Allo	No	Behavioral improvement<br>Decreased dopaminergic neuronal loss was detected<br>MSCs *in vivo* differentiation status not reported	135

PD Parkinson’s Disease, MSCs Mesenchymal stromal/stem cells, 6-OHDA 6-hydroxydopamine, BM bone marrow, AD adipose tissue, UC umbilical cord, MPTP 1-methyl-4-phenyl-1,2,3,6-tetrahydropyridine, Auto autologous, Allo allogenic
